# Heteroresistance to piperacillin/tazobactam in *Klebsiella pneumoniae* is mediated by increased copy number of multiple β-lactamase genes

**DOI:** 10.1093/jacamr/dlae057

**Published:** 2024-04-10

**Authors:** Ahmed Babiker, Sarah Lohsen, Julia Van Riel, Karin Hjort, David S Weiss, Dan I Andersson, Sarah Satola

**Affiliations:** Division of Infectious Diseases, Department of Medicine, Emory University School of Medicine, Atlanta, GA, USA; Division of Infectious Diseases, Department of Medicine, Emory University School of Medicine, Atlanta, GA, USA; Division of Infectious Diseases, Department of Medicine, Emory University School of Medicine, Atlanta, GA, USA; Department of Medical Biochemistry and Microbiology, Uppsala University, Uppsala, Sweden; Division of Infectious Diseases, Department of Medicine, Emory University School of Medicine, Atlanta, GA, USA; Department of Medical Biochemistry and Microbiology, Uppsala University, Uppsala, Sweden; Division of Infectious Diseases, Department of Medicine, Emory University School of Medicine, Atlanta, GA, USA

## Abstract

**Background:**

Piperacillin/tazobactam is a β-lactam/β-lactamase inhibitor combination with a broad spectrum of activity that is often used as empirical and/or targeted therapy among hospitalized patients. Heteroresistance (HR) is a form of antibiotic resistance in which a minority population of resistant cells coexists with a majority susceptible population that has been found to be a cause of antibiotic treatment failure in murine models.

**Objectives:**

To determine the prevalence of HR and mechanisms of HR to piperacillin/tazobactam among *Klebsiella pneumoniae* bloodstream infection (BSI) isolates.

**Materials:**

From July 2018 to June 2021, *K. pneumoniae* piperacillin/tazobactam-susceptible BSI isolates were collected from two tertiary hospitals in Atlanta, GA, USA. Only first isolates from each patient per calendar year were included. Population analysis profiling (PAP) and WGS were performed to identify HR and its mechanisms.

**Results:**

Among 423 *K. pneumoniae* BSI isolates collected during the study period, 6% (25/423) were found to be HR with a subpopulation surviving above the breakpoint. WGS of HR isolates grown in the presence of piperacillin/tazobactam at concentrations 8-fold that of the MIC revealed copy number changes of plasmid-located β-lactamase genes *bla*_CTX-M-15_, *bla*_SHV33_, *bla*_OXA-1_ and *bla*_TEM-1_ by tandem gene amplification or plasmid copy number increase.

**Conclusions:**

Prevalence of HR to piperacillin/tazobactam among bloodstream isolates was substantial. The HR phenotype appears to be caused by tandem amplification of β-lactamase genes found on plasmids or plasmid copy number increase. This raises the possibility of dissemination of HR through horizontal gene transfer and requires further study.

## Introduction

Piperacillin/tazobactam is a β-lactam/β-lactamase inhibitor combination with a broad spectrum of activity that is often used as empirical and/or targeted therapy among hospitalized patients.^1^ A recent randomized trial suggested an increased risk of mortality for ceftriaxone non-susceptible Enterobacterales infections treated with piperacillin/tazobactam compared with meropenem, despite MICs within the susceptible range, calling into question its use in such infections.^2^ Heteroresistance (HR), is a resistance phenotype where, in a main population of susceptible cells, a minority population of resistant cells exists that may cause antibiotic treatment failure.^3^ Here, we determined the prevalence and mechanisms of HR to piperacillin/tazobactam among clinical *Klebsiella pneumoniae* bloodstream infection (BSI) isolates.

## Materials

Isolate inclusion

*K. pneumoniae* BSI isolates from July 2018 to June 2021 were prospectively collected from two tertiary care hospitals in Atlanta, GA, USA. Isolates underwent antimicrobial susceptibility testing (AST) as part of routine clinical care on the VITEK 2 (bioMérieux, Durham, NC, USA). Isolates susceptible to piperacillin/tazobactam based on CLSI breakpoints (≤16 mg/L) were selected.^4^ Only the first isolate from each patient per calendar year was included.

HR population analysis profiling (PAP)

PAP testing for HR to piperacillin/tazobactam was conducted per a modified protocol of the microdilution plating method as previously described.^5,6^ For HR determination, log-transformed ratios of cfu/mL of colonies surviving on drug relative to cfu/mL without the presence of drug were calculated at the breakpoint. Strains were considered HR if they displayed a log-transformed ratio (cfu/mL growing on the antibiotic breakpoint plate divided by the cfu/mL in the starting inoculum with no antibiotic) of less than −0.3 (less than 50% survival) but above the limit of detection (i.e. survival ≥10^−6^). All PAPs were performed in duplicate and isolates that were identified as HR were further validated.

Selection of resistant mutants at 8-fold MIC

Two separate colonies grown on Mueller–Hinton (MH) plates were inoculated into 1 mL of CAMHB from the different parental HR isolates (Table 1) and incubated overnight at 37°C with shaking at 180 rpm. From each overnight culture, 100 µL was spread on MH agar plates containing piperacillin/tazobactam (piperacillin:tazobactam ratio 8:1) at 8-fold MIC. Colonies from each plate were single-cell purified by restreaking on plates with the same piperacillin/tazobactam concentration. One random colony from each plate was inoculated into 1.5 mL of MH broth with addition of piperacillin/tazobactam (at 8-fold MIC) and grown overnight at 37°C. From each bacterial culture a frozen stock and a cell pellet (from 500 µL culture) were saved for DNA extraction and WGS. MICs for the HR strains and selected mutants were determined using ETEST (bioMérieux).

**Table 1.** WGS of HR strains and resistant mutant subpopulations

Parental HR isolate				Mutant isolated at 8-fold MIC								
Strain DA# (isolate)	MIC TZP (mg/L)	*bla* genes		Mutant DA#	MIC TZP (mg/L)	Mutations	Tandem amplification				PCN	
		Chromosome	Plasmid (kb)				Amplified *bla* gene	Fold amplification	Size of amplification (kb)	Amplification endpoints	PCN increase	Amplified *bla* gene
DA76126 (K13)	2	*bla*_SHV-11_	*bla*_CTX-M-15_, *bla*_TEM-1_ (137)	DA79094	64	IS insertion in the *bla*_SHV-11_ promoter						
DA76135 (K399)	16	*bla*_SHV-1_	*bla*_SHV-33_ (217)	DA79100	>256	No mutations	*bla*_SHV-33_	26×	8.3	IS*26*		
DA76136 (K451)	8	*bla*_SHV-1_	*bla*_OXA-1_ (138)	DA79105	96	No mutations	*bla*_OXA-1_	4.5×	18.7	IS*26*		
DA76139 (K618)	8	*bla*_OKP-A12_ (SHV-1)	*bla*_TEM-1_ (234, 147)^a^	DA79106	48	No mutations					35×	*bla*_TEM-1_
											13×	*bla*_TEM-1_
DA76144 (K833)	16	*bla*_SHV-28_	*bla*_CTX-M-15_, *bla*_OXA-1_, *bla*_TEM-1_ (242)	DA79110	≥24^b^	No mutations	*bla*_CTX-M-15_, *bla*_OXA-1_	2×	39.7	IS element		

PCN, plasmid copy number; TZP, piperacillin/tazobactam.

^a^Two plasmids with *bla*_TEM-1_.

^b^This mutant showed a mixed population with subpopulations of cells with MIC ≥24 mg/L.

### WGS of parental isolates and selected mutants

DNA was extracted from the resistant mutants and the respective five parental HR isolates using the MasterPure Gram Negative DNA Extraction Kit (Lucigen, Middleton, WI, USA) (Table 1). Parental HR isolates underwent both long- and short-read sequencing, while resistant mutants underwent short-read sequencing only (Supplementary Materials, available as Supplementary data at *JAC-AMR* Online). To estimate the level of DNA amplification, the average sequence coverage of the amplified β-lactamase (*bla*) gene was divided by the average sequence coverage of sequences outside the amplified region on the plasmid or chromosome using the CLC Genomic Workbench. Similarly, the increase in plasmid copy number was determined by dividing the sequence coverage of the plasmid with that of the chromosome. For the calculation of the average sequence coverage of the plasmid DNA sequence, any amplification on the plasmid was removed and the average sequence coverage was recalculated for the remaining plasmid sequence. The Comprehensive Antibiotic Resistance Database (CARD) online database was used to identified resistance genes, using the preset selection criteria (strict cut-off hits with an identity percentage of reference sequence >90% and a percentage length of reference sequence >90%).^7^

### Statistical analysis

Differences in proportion of HR isolates by MIC category were assessed by Pearson’s chi-squared test with Yates’ correction. Statistical analysis and figure generation was performed on R using RStudio v 2023.09.1 + 494 (R Foundation for Statistical Computing, Vienna, Austria).

### Ethics

The data collection and analysis were approved by the Emory University Institutional Review Board (IRB#00093057).

## Results

**Figure 1.**
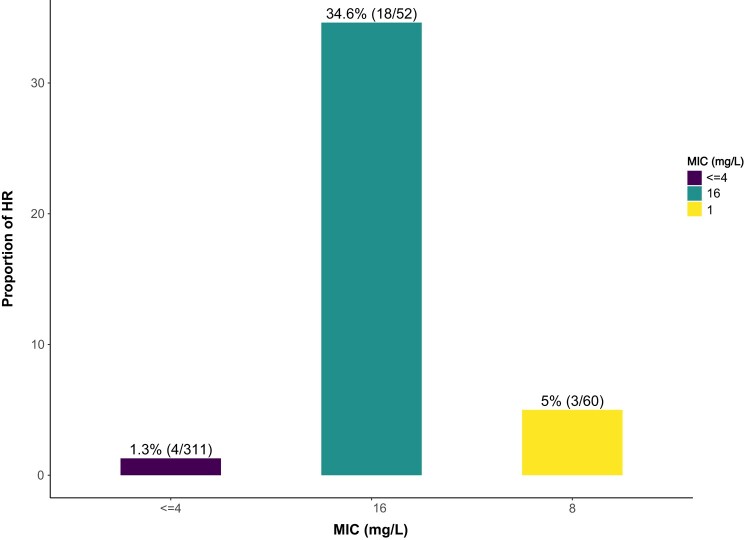
Prevalence of HR by MIC. MIC determined by testing on the VITEK 2 (bioMérieux) as part of routine clinical care.

Four hundred and twenty-three *K. pneumoniae* BSI isolates were collected during the study period. Of these, 310/423 (73.3%) had no growth above the breakpoint concentration of piperacillin/tazobactam on pre-screen and were not selected for a PAP. Among the remaining 113 selected for PAP, 25/423 (6%) were found to be HR. Of the HR isolates, 4/25 (16%) had an MIC of ≤4 mg/L, 3/25 had an MIC of 8 mg/L and 18/25 (72%) had an MIC of 16 mg/L. The proportion of HR isolates differed significantly by MIC category (*P* < 0.001) with an increased baseline MIC (Figure 1). Fifteen of the 25 HR isolates (60%) were resistant to ceftriaxone and presumptively considered ESBL producers as per CLSI guidance. None of the isolates were carbapenem resistant.

To identify the mechanism behind HR to piperacillin/tazobactam in the *K. pneumoniae* isolates, five HR strains and selected resistant mutant subpopulations underwent WGS (Table 1). Among the HR isolates analysed, an increased copy number of resistance genes was the most common cause for generating the resistant subpopulation. Four out of five parental isolates and their corresponding resistant subpopulations displayed increased copy number of different *bla* genes. The specific genetic changes involved either increased plasmid copy number or tandem amplifications of *bla* genes located on plasmids. For the parental isolate DA76139, an increase in the copy number, 13-fold and 35-fold, was found for two different plasmids p148 and p234, respectively, both present in this parental isolate and both containing the *bla*_TEM-1_ gene. Resistant subpopulations isolated from the parental isolates DA76135, DA76136 and DA76144 contained tandem amplifications of the *bla*_OXA-1_, *bla*_CTX-M-15_ and *bla*_SHV-33_ genes present on plasmids, either singly or in combination, with copy numbers increases ranging from 2- to 26-fold (Table 1). Finally, one isolate (DA76126) showed a mutational resistance mechanism where an IS*1380* (IS*Ecp1*) element had jumped into the promoter region of the *bla*_SHV-11_ gene located on the chromosome.

## Discussion

Our study is one of the first to systematically estimate the prevalence of HR to piperacillin/tazobactam, among *K. pneumoniae* BSI isolates. Moreover, comparative genomic approaches revealed important insights into the mechanism of HR and found that the HR phenotype was generated by tandem amplification of *bla* genes found on plasmids or plasmid copy number increases.

The continued surveillance of HR is crucial as it often goes undetected by routine AST methods and may lead to clinical failure.^3,8^ Our study is one of the first to systematically estimate the prevalence of HR among clinical BSI isolates and builds on the small sample sizes of other studies.^9–13^ We found the prevalence of HR to be considerable among isolates with a susceptible MIC, with HR more likely among isolates with a higher baseline MIC. Although the impact on clinical outcomes remains unclear,^14–16^ animal and some clinical studies have implicated HR in antibiotic failure.^8^ Thus, ongoing surveillance for HR is warranted, as is continued investigation into its impact on clinical outcomes.

The two most common mechanisms of HR described include: (i) mutational HR, where the resistant subpopulation of cells carry unstable classical resistance mutations; and (ii) increased dosage of resistance genes either via tandem amplification and/or increased plasmid copy number.^3, 9^ Previous studies have implicated mobile genetic elements (MGEs) in providing tandem repeat sequences for amplification of narrow-spectrum β-lactamases, such as piperacillin/tazobactam resistance.^10–13^ Our study of piperacillin/tazobactam-susceptible HR isolates reveals that similar gene amplification contributes to the development of antibiotic resistance. MGE-mediated *bla* gene amplifications may also confer more concerning phenotypes such as carbapenem resistance.^17,18^ Moreover, HR-implicated ISs such as IS26 have been associated with co-location of other AMR genes^11^ and with the transfer of carbapenemase (CP) genes,^19^ raising the possibility of resistance to novel β-lactam/β-lactamase inhibitors and non-β-lactam antimicrobials through gene amplification.^3,9^ Whether the amplifications studied here are stable in the absence of drug exposure is yet to be determined.^9,11,18,20^ Currently, syndromic multiplex rapid diagnostics, which have become an integral part of clinical care in the USA, only include ESBL and CP genes.^21^ Given their potential implications in driving piperacillin/tazobactam HR and resistance phenotypes upon antibiotic exposure, inclusion of narrow-spectrum *bla* genes as important genotypic markers should be considered.

Given its broad-spectrum Gram-negative activity, piperacillin/tazobactam is a common choice for empirical and definitive therapy among hospitalized patients.^1,22^ In the landmark MERINO trial, piperacillin/tazobactam was found to be not non-inferior to meropenem for the treatment of piperacillin/tazobactam-susceptible Enterobacterales BSIs.^2^ These findings led to recommendations to avoid piperacillin/tazobactam for the treatment of infections outside of the urinary tract caused by ESBL-producing Enterobacterales, even if susceptibility is demonstrated.^23^ A *post hoc* analysis of the MERINO trial found that many susceptible isolates with raised piperacillin/tazobactam MICs harboured narrow-spectrum *bla* genes such as *bla*_OXA-1_.^24^ Exposure of these potential HR strains to piperacillin/tazobactam may have led to amplification of β-lactamases, leading to clinical failure. Further prospective clinical studies that incorporate MIC testing, characterization of HR status, *bla* gene content and therapeutic drug monitoring are needed to untangle the potential role of HR in driving clinical outcomes and optimize therapeutic strategies.

The potential for clinically important discrepancies when performing piperacillin/tazobactam AST testing was evident in the MERINO clinical trial and subsequent *post hoc* analysis, where a number of isolates originally considered susceptible at local sites were confirmed to be resistant after retesting at the central laboratory.^25,26^ Such methodological inconsistencies can lead to reporting of falsely low MICs. This issue is compounded by the correlation of increased mortality with higher piperacillin/tazobactam MIC levels,^1^ which supports the decision of CLSI to revise the piperacillin/tazobactam susceptible breakpoint to ≤8/4 mg/L.^26^ However, there is significant variability in adopting revised breakpoints among clinical laboratories.^27^ This, along with abovementioned testing issues, may have a significant impact on clinical care.^24^ Moreover, as HR definitions are dependent on breakpoints and MICs as benchmarks, AST challenges and poor uptake of updated breakpoints can significantly impact HR estimates. Our study was done using 2021 breakpoints, thus our reported HR prevalence may be an underestimation.

Our study has some limitations. Despite our robust methodology, reported rates of HR may have been underestimated as transient HR, particularly in cases where spontaneous unstable gene amplification may not have been captured.^9^ We excluded repeat isolates from the same patient; however, HR may be more likely to be detected in these patients given likely antibiotic exposure.^18^ Our study was a single-centre study and hence our estimated HR prevalence may not be applicable to other geographical locations. Moreover, as only a subset of randomly selected isolates underwent WGS, our mechanistic findings may not be applicable to all HR isolates.

In conclusion, we found prevalence of HR to piperacillin/tazobactam among bloodstream isolates substantial and often mediated by tandem amplification of *bla* genes associated with MGEs or increased plasmid copy number. This raises the possibility of dissemination of HR through antibiotic pressure and horizontal gene transfer. Continued surveillance and clinical studies of HR is paramount to assess its role in treatment failure and contribution to the emergence of resistant strains.

## Supplementary Material

dlae057_Supplementary_Data

## Data Availability

Genome sequences used in the analysis are available in the sequence read archive under BioProject PRJNA1065253.

## Supplementary data

Supplementary material available as Supplementary data at *JAC-AMR* Online.
