# Supplementary material for: Heteroresistance to piperacillin/tazobactam in Klebsiella pneumoniae is mediated by increased copy number of multiple β-lactamase genes
Source: JAC Antimicrob Resist. 2024 Apr 10;6(2):dlae057. doi: 10.1093/jacamr/dlae057 (PMC11004786; doi:10.1093/jacamr/dlae057)
Supplement: dlae057_Supplementary_Data [file dlae057_supplementary_data.docx]

**Supplementary Material**

**Materials:**

Heteroresistance Population Analysis Profiling:

Bacterial strains were streaked from frozen glycerol stocks onto Mueller-Hinton (MH) II plates and grown overnight at 37ºC. First, isolates underwent a pre-screen for selection for population analysis profiling (PAP) by plating 100 mL of 10^8^ CFU/mL from 2 biological replicates grown overnight at 37°C and plated in triplicate on a MH II plates with 16/4 mg/ml of piperacillin-tazobactam (TZP) for isolates with an MIC ≤ 8/4mg/ml or on plates with 32/4 µg/mL of TZP for isolates with an MIC of 16 mg/ml of TZP depending on isolate clinical MIC. Plates were examined at 24 and 48 hours and if at least one colony forming unit (CFU) was found the isolate was selected for PAP. Per CLSI standards for antibiotic susceptibility testing (AST), we sub-cultured each isolate from the freezer stock twice prior to testing by PAP for heteroresistance (HR). Single colonies were selected to inoculate a 48-well plate containing 300 µl of cation-adjusted Mueller-Hinton broth (CaMHB), and cultures were grown for approximately 18 hours at 37ºC in a shaking incubator at 500 rpm. Cultures were serially diluted 1:10 in CaMHB over a series of seven dilutions. 7.5 µl of the dilutions were spotted on MH II agar plates with no antibiotic as well as 2-fold increasing concentrations of antibiotics. The following concentrations of antibiotic were used for: 0.025/4, 0.5/4, 1/4, 2/4, 4/4, 8/4, 16/4, 32/4 and 64 mg/L. Plates were incubated for 20-24 hours at 37ºC before colonies were counted and again at 48 hours. For HR determination, log-transformed ratios of CFUs/mL of colonies surviving on drug relative to CFUs/mL without the presence of drug were calculated at the breakpoint. Strains were considered HR if they displayed a log transformed ratio (CFU/mL growing on the antibiotic breakpoint plate divided by the CFU/mL in the starting inoculum with no antibiotic) of less than –0.3 (less than 50% survival) but above the limit of detection (usually more than 0.0001% survival). All PAPs were performed in duplicate and isolates that were identified as HR were validated a second time.

Whole genome sequencing of parental isolates and selected mutants: DNA was extracted from the resistant mutants and the respective five parental HR isolates using the MasterPure Gram Negative DNA Extraction Kit (Lucigen) (**Table 1**). Parental HR isolates underwent long and short read sequencing. Given the parent strains were already characterized using both long and short read sequencing the subsequent generated mutations were sequenced with short read short-read sequencing only. This was done Nanopore (Oxford Nanopore Technologies, United Kingdom) was done in-house on the MinION Mk1C using the rapid barcoding kit 96 to multiplex 40 genomes per each R9 cells. Short reads were generated with DNBseq (on average 50x coverage and 800 base pairs paired-end libraries) by BGI (Warsaw, Poland). The quality of sequencing reads was validated using FastQC v0.11.91 and MultiQC v1.122. Short reads were trimmed using fastp v0.20.13. For Nanopore, reads shorter than 1000 bp were removed using Filtlong v.0.2.14. Sequenced and filtered reads were assembled using Unicycler v0.4.85 if Nanopore coverage was below 20x; otherwise, a custom pipeline involving Flye v2.9.16, medaka v1.6.07, Polypolish v0.5.08, BWA v0.7.17 (43), and seqkit v2.2.0 was utilized. To annotate tRNA and protein-coding genes, Prokka v1.14.69 and Prodigal v2.6.310, along with tRNA scan-SE v2.0.911 was used. The DNBseq reads from the selected clones were mapped onto their corresponding parental HR isolate. Mutations and amplifications were detected using basic variant detection, InDels, structural variants and coverage analysis with the CLC software (Qiagen).
